# Infectious Agents & Cancer reviewer acknowledgement 2012

**DOI:** 10.1186/1750-9378-8-5

**Published:** 2013-04-18

**Authors:** Franco M Buonaguro, Sam M Mbulaiteye

**Affiliations:** 1 Istituto Nazionale Tumori, Naples, Italy; 2 National Cancer Institute, Bethesda, USA

## Abstract

**Contributing reviewers:**

The editor of *Infectious Agents & Cancer* would like to thank all our reviewers who have contributed to the journal in Volume 7 (2012).

## 

Clement Adebamowo

United States of America

Francisco Aguayo

Chile

Nosheen Ahmed

Pakistan

Oluyemisi Akinwande

Nigeria

Doglio Alain

France

Clorinda Annunziata

Italy

Luigi Aurisicchio

Italy

Athanase Billis

Brazil

Patrizia Bonelli

Italy

Christal Bowman

United States of America

Sabine Brandt

Austria

Michele Caraglia

Italy

Daniel Carr

United States of America

Chiara Cattaneo

Italy

Leonidas Chelis

Greece

Jianming Chen

United States of America

Paolo Chieffi

Italy

Manola Comar

Italy

Volker Daniel

Germany

Laura De Marco

Italy

Valli De Re

Italy

Silvia de Sanjose

Spain

Riccardo Dolcetti

Italy

Antonina Dolei

Italy

Geraldina Dominguez

United States of America

Ryan Dowling

Canada

Charles Dzamalala

Malawi

Sherine Elsawa

United States of America

Atef Fahim

Egypt

Heba Fawzy

Egypt

Weiwei Feng

China

Seyed-Mohammad Fereshtehnejad

Iran

Silvia Franceschi

France

Renato Franco

Italy

Israel Franco

United States of America

Nancy Gasper-Smith

United States of America

Paolo Giorgi Rossi

Italy

James Goedert

United States of America

Henri Gruffat

France

Mohamed Hafez

Egypt

Chang Haixin

China

Sylvie Hermouet

France

Myfanwy Hopkins

United States of America

Colin Hopper

United Kingdom

John Idoko

Nigeria

RS Jayshree

India

Anbu Karani-Adikesavan

United States of America

Charles Kilewo

Tanzania

James Kitinya

Tanzania

Vijay Kumar

India

Eduardo Lazcano-Ponce

Mexico

Feng Li

United States of America

Wenjin Liu

United States of America

Vicente Madrid-Marina

Mexico

Rita Mancini

Italy

Maureen Martin

United States of America

Muhammad Shareef Masoud

Pakistan

Tanja Matijevic Glavan

Croatia

Alicia McDonald

United States of America

Hashim Missawi

Sudan

Sulma Mohammed

United States of America

Hector Montoya-Fuentes

Mexico

Olivier Morales

France

Denis Mottet

Belgium

George Mutter

United States of America

Ariela Noy

United States of America

Luigi Panico

Italy

Patrizia Pasanisi

Italy

Daniela Pasquali

Italy

Annacarmen Petrizzo

Italy

Beatriz Pogo

United States of America

Maurizio Provenzano

Switzerland

Peter Rambau

Tanzania

Hervé Reychler

Belgium

Paramananda Saikia

India

Lars Sand

Sweden

Sreenivasan Sasidharan

Malaysia

Wellerson Scarano

Brazil

Michael Selgrad

Germany

Renat Shaykhiev

United States of America

Abdul Moid Shehzad

Pakistan

Misuzu Shimakage

Japan

Laura Sichero

Brazil

Freddy Sitas

Australia

Heath Skinner

United States of America

Elaine Smith

United States of America

Gamil Tawadrous

Egypt

Mojtaba Teimoori

Iran

Frederic Thomas

France

Andrea Tinelli

Italy

Maria Lina Tornesello

Italy

Helen Trottier

Canada

Gooitzen van Dam

Netherlands

Aldo Venuti

Italy

Maria Luisa Visciano

Italy

Giovanni Vitale

Italy

Elisabete Weiderpass

Norway

Jianfei Xue

United States of America

Yibin Yang

United States of America

Anna Linda Zignego

Italy

